# A 55-Year-Old Male Presenting With a Lower Extremity Rash: A Case of Immunoglobulin A (IgA) Nephropathy

**DOI:** 10.7759/cureus.14165

**Published:** 2021-03-29

**Authors:** Eric Denha, Ali Rahim, Sunjay Modi, Oghenekpaobor Oyibo, Megan Scott

**Affiliations:** 1 Internal Medicine, Henry Ford Health System, Detroit, USA; 2 Anaesthesiology, Henry Ford Health System, Detroit, USA

**Keywords:** iga nephropathy, hepatitis c, leukocytoclastic vasculitis

## Abstract

Immunoglobulin A (IgA) nephropathy, mesangial deposition of IgA in renal parenchyma, typically presents with hematuria and proteinuria. Leukocytoclastic vasculitis (LCV), a small-vessel vasculitis, can present secondary to IgA. We will discuss a case of secondary IgA nephropathy with concomitant LCV in a patient with reactivated hepatitis C. A 55-year-old male with decompensated alcoholic cirrhosis presented for a bilateral lower-extremity rash. The patient was diagnosed with IgA nephropathy, by kidney biopsy, and skin biopsy showing LCV. Further investigation revealed hepatitis C viral load was 275,000. We present a rare presentation of secondary IgA nephropathy with concomitant LCV, which we hypothesize was secondary to reactivation of hepatitis C.

## Introduction

Immunoglobulin A (IgA) nephropathy may present in two forms, either primary or secondary, and both are associated with mesangial deposition of IgA. Clinical features of IgA nephropathy may include gross or macroscopic hematuria, impaired kidney function evidenced by decreased glomerular filtration rate (GFR), elevated creatinine, or proteinuria (seen in advanced disease). Secondary IgA nephropathy is usually associated with liver cirrhosis, as seen in our patient who presented with a reactivation of hepatitis C [1]. Leukocytoclastic vasculitis, a type of small-vessel vasculitis, is an inflammatory reaction composed of neutrophils that undergo breakdown and release nuclear debris. Etiologies may include idiopathic, systemic vasculitis (such as IgA in our patient), bacterial infections, drug-induced, or malignancy.

## Case presentation

**Figure 1:**
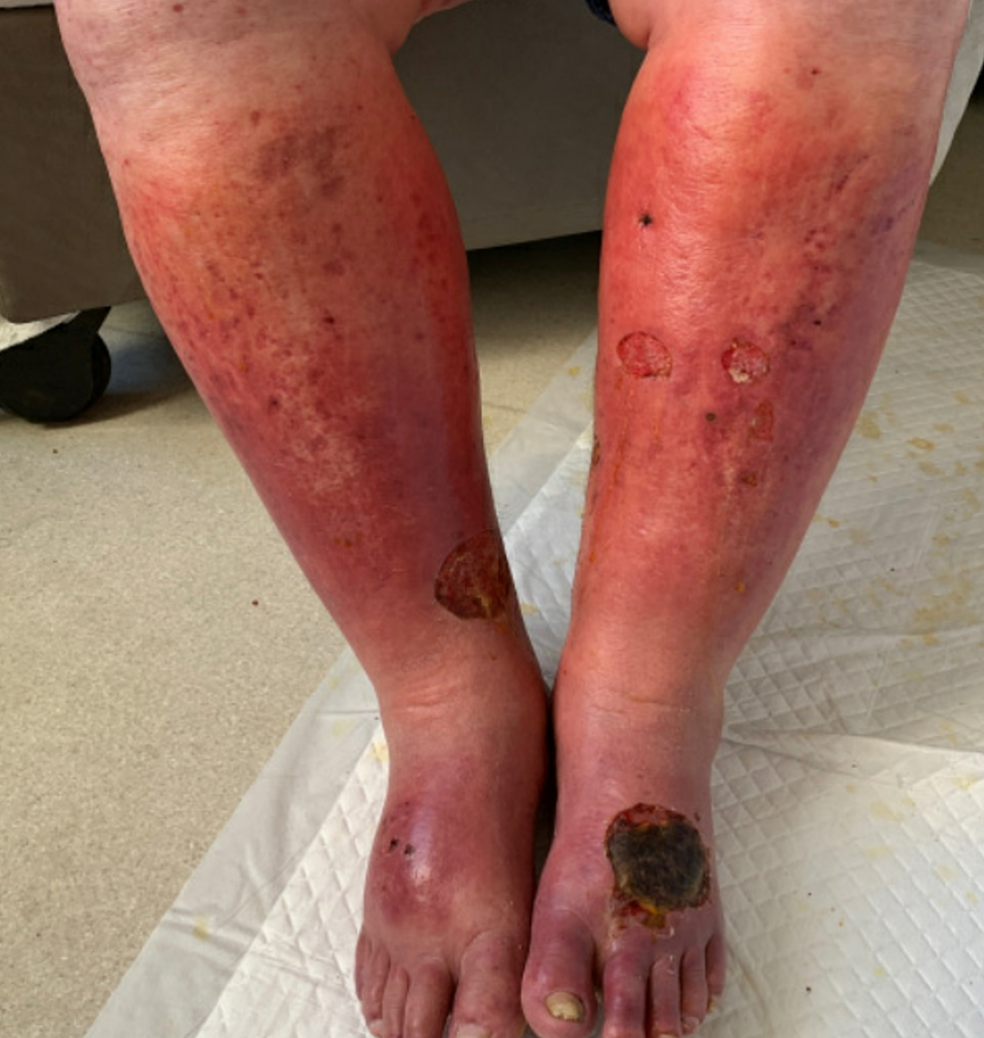
Photo of Patient's Bilateral Lower Extremity Rash

Our patient is a 55-year-old male with a past medical history of alcoholic cirrhosis complicated by varices status post transjugular intrahepatic portosystemic shunt (TIPS), hepatitis C (treated with ledipasvir/sofosbuvir), mesenteric vein thrombosis, and microscopic hematuria, who presented from outside hospital for worsening kidney function and bilateral lower extremity rash (Figure 1). His liver enzymes were elevated (Alanine transaminase [ALT] 113 IU/L, aspartate transaminase [AST] 163 IU/L) with total bilirubin elevation to 1.7 mg/dL. An acute hepatitis panel was obtained and was negative, however, hepatitis C antibody was positive and viral load was 275,000. His creatinine was elevated to 7.3 mg/dL from baseline 1.5-1.7 mg/dL, with an albumin:creatinine ratio of 5,581. An extensive workup was performed including an autoimmune panel, which was negative for antinuclear antibody (ANA), perinuclear antineutrophil cytoplasmic antibody (p-ANCA), cytoplasmic antineutrophil cytoplasmic antibody (c-ANCA) and CT kidneys that showed no hydronephrosis. A CT-guided renal biopsy performed and showed IgA nephropathy. Serum cryoglobulin was obtained and was positive; his complement levels were low. Our patient developed a worsening rash two weeks prior to hospitalization on his bilateral legs, associated with swelling and pain. Biopsy performed at outside hospital was consistent with leukocytoclastic vasculitis without IgA. Over the course of the hospitalization, the patient’s kidney function and leukocytoclastic vasculitis improved with conservative management. The patient was discharged with hepatology follow-up for initiation of sofosbuvir/velpatasvir and potential initiation of angiotensin-converting enzyme (ACE) inhibitor/angiotensin receptor blocker (ARB) to prevent worsening kidney function [2].

## Discussion

Primary IgA nephropathy is known to be the main cause of primary glomerulonephritis, typically seen in male patients in their 20s-30s. These patients (40-50%) typically present with gross hematuria and recurrent sinopulmonary infections. IgA nephropathy, however, is also associated with other conditions, such as cirrhosis and HIV infections. Our patient likely had secondary IgA nephropathy from his reactivation of hepatitis C with superimposed cirrhosis. Just like primary IgA nephropathy, a kidney biopsy is needed to diagnose secondary nephropathy. Pathogenesis behind cirrhosis-induced IgA nephropathy involves the inability of impaired Kupffer cells to remove IgA-containing complexes. We hypothesize that the leukocytoclastic vasculitis was due to IgA nephropathy which was seen on kidney biopsy, even though this patient had elevated cryoglobulins [3]. The treatment for such would be focusing on the underlying condition, in this case treating the reactivated hepatitis C. Systemic steroids, sometimes used to treat primary IgA nephropathy, would be contraindicated as it would worsen this patient’s kidney dysfunction and not effectively be able to treat his skin rash which is related to IgA vasculitis, as seen in prior studies [4]. In fact, in 90% of the cases, a rash secondary to IgA vasculitis will resolve spontaneously. Prior studies have found that treatment of hepatitis C with interferon (INF)-alpha has shown improvement in IgA nephropathy. A reported case by Dey et al. showed improvement in proteinuria with INF-alpha therapy targeted against hepatitis C [5]. The addition of ACE inhibitor or ARB therapies, however, will be important in preserving these patients’ kidney function, as seen in a study by Praga et al. in which 44 IgA nephropathy patients with proteinuria and baseline creatinine of <1.5 mg/dl were randomly assigned to receive enalapril or other anti-hypertensives than ACE/ARB [2]. Patients in the enalapril group showed a significant improvement in renal survival, defined as a less than fifty percent rise in serum creatinine after six years [2].

## Conclusions

We discussed a case of secondary IgA nephropathy in a patient with reactivated hepatitis C. Our 55-year-old patient was diagnosed with IgA nephropathy, by kidney biopsy, and skin biopsy showing leukocytoclastic vasculitis. We conclude that the leukocytoclastic vasculitis was due to IgA nephropathy, although skin biopsy did not show any IgA deposition. Treatment in this case would be focusing on the patient’s underlying condition - hepatitis C. What needs to be further addressed in this population is the use of both ACE inhibitors and ARBs in preserving renal function.
